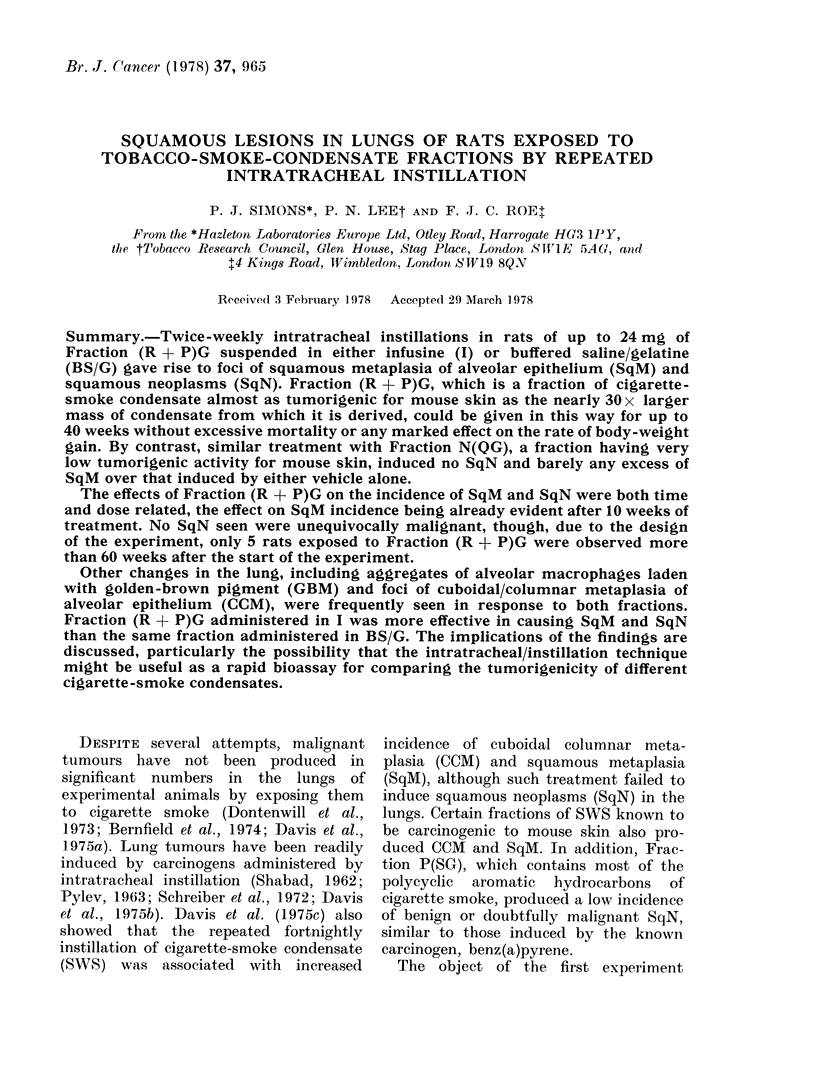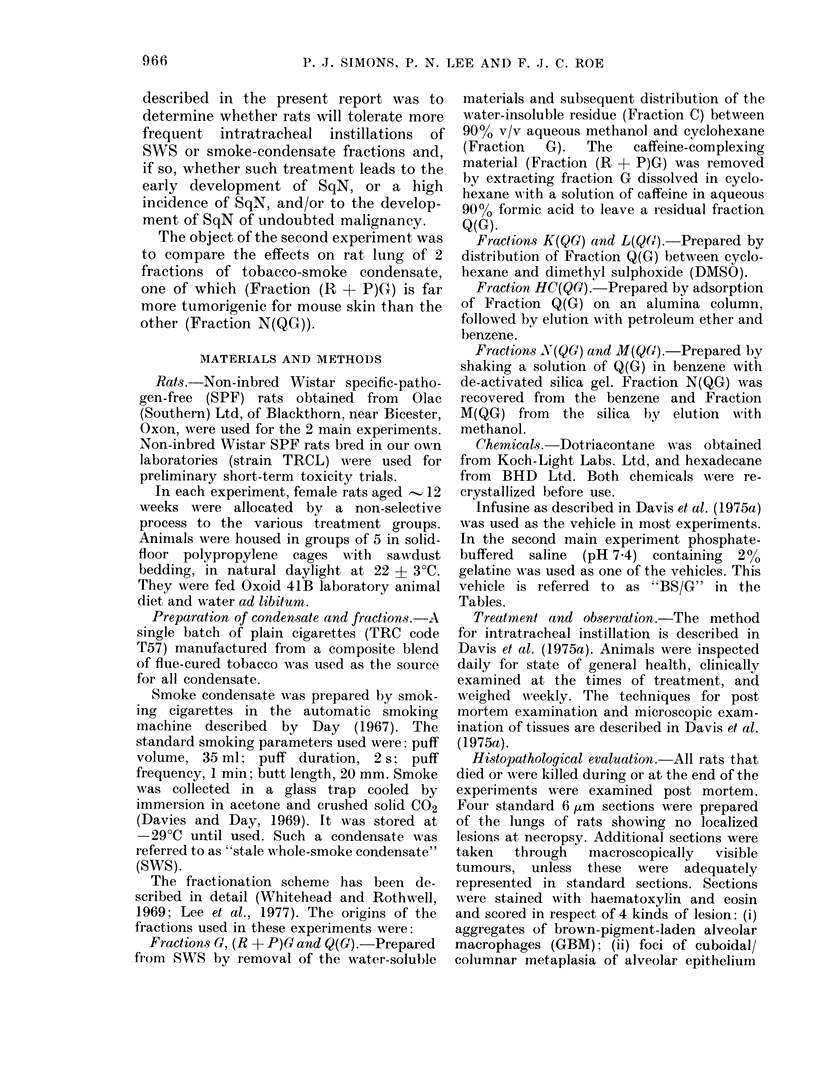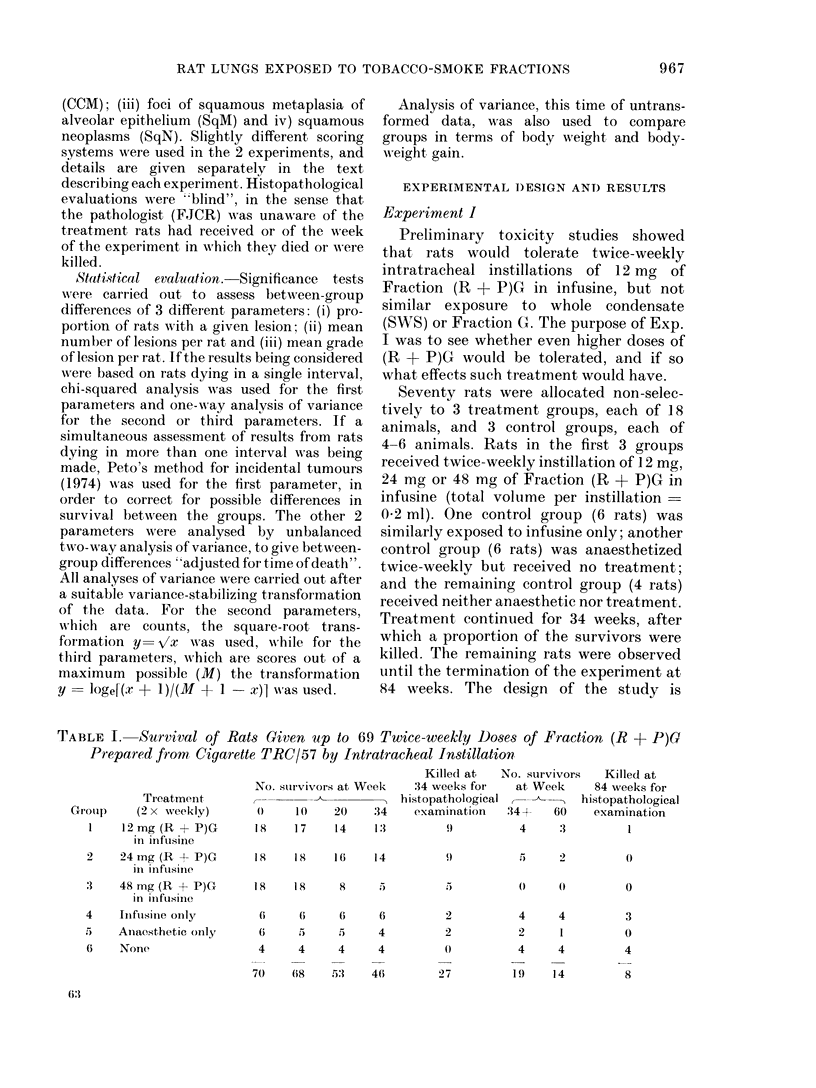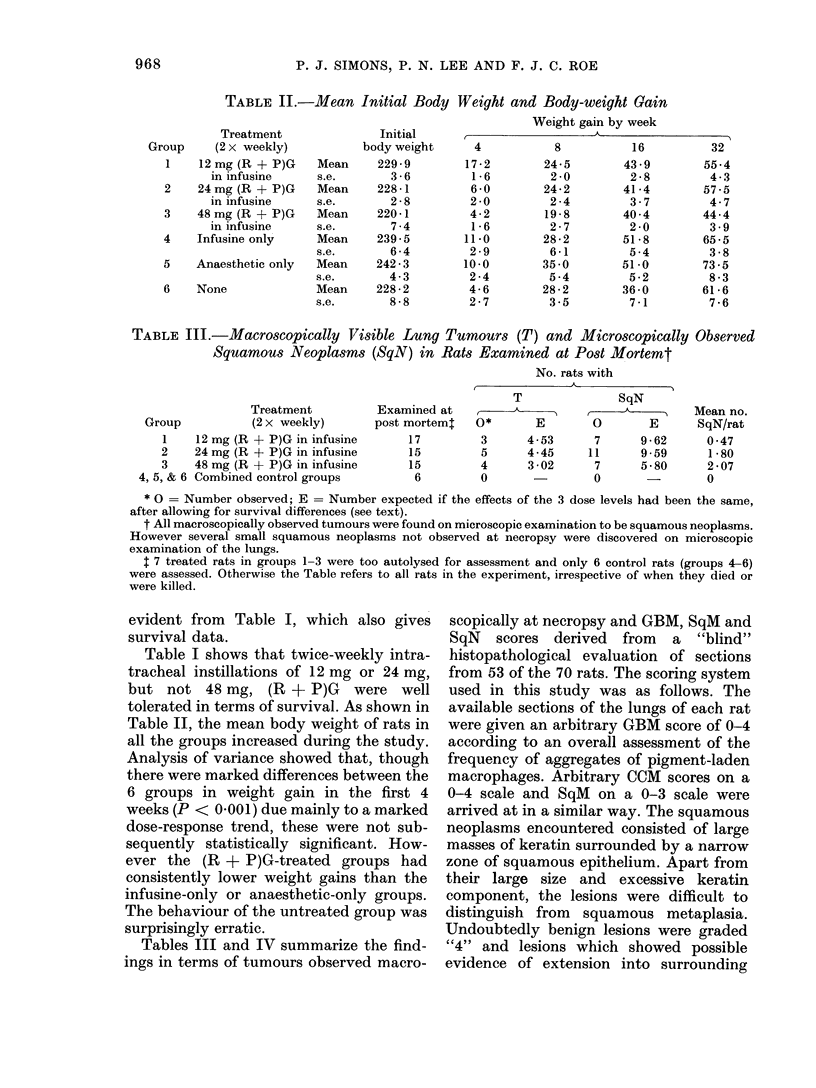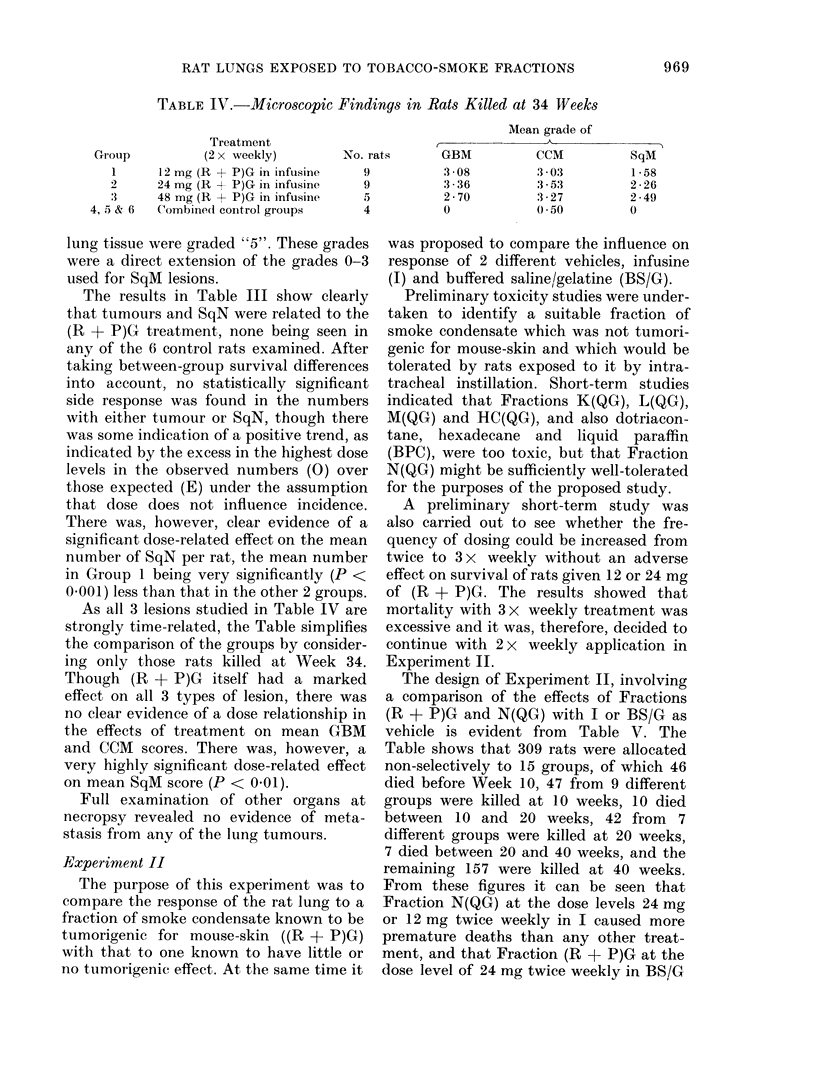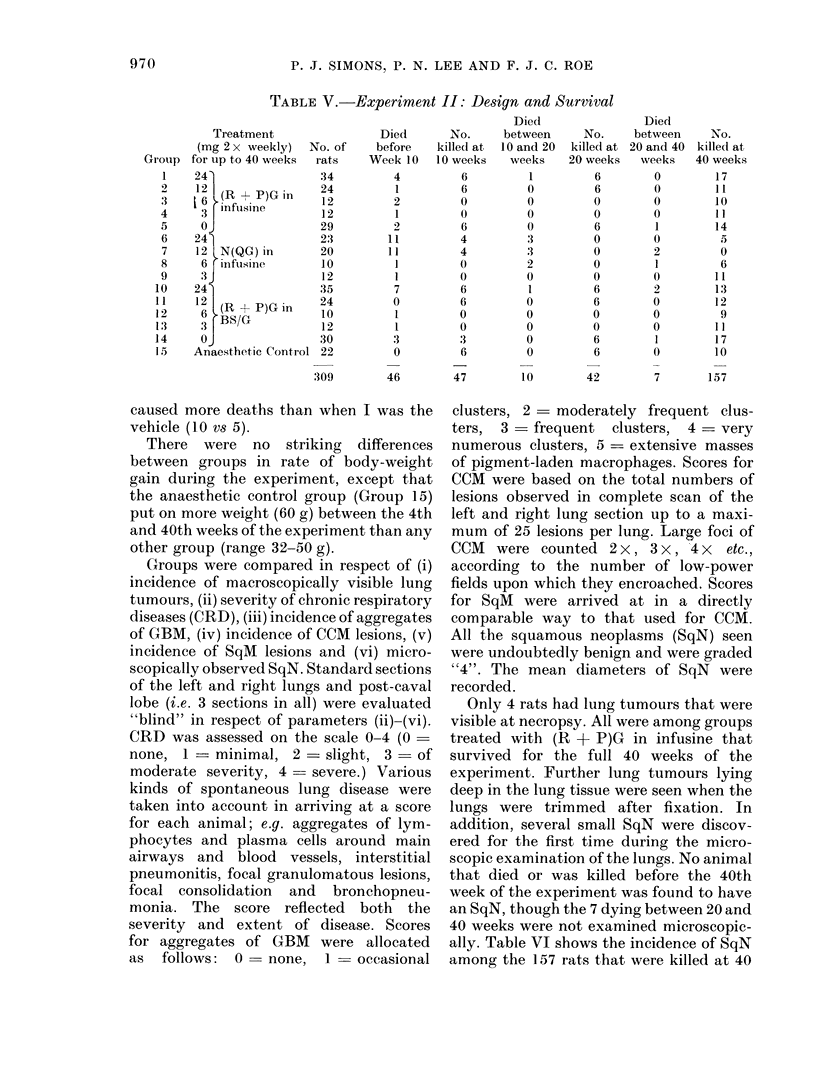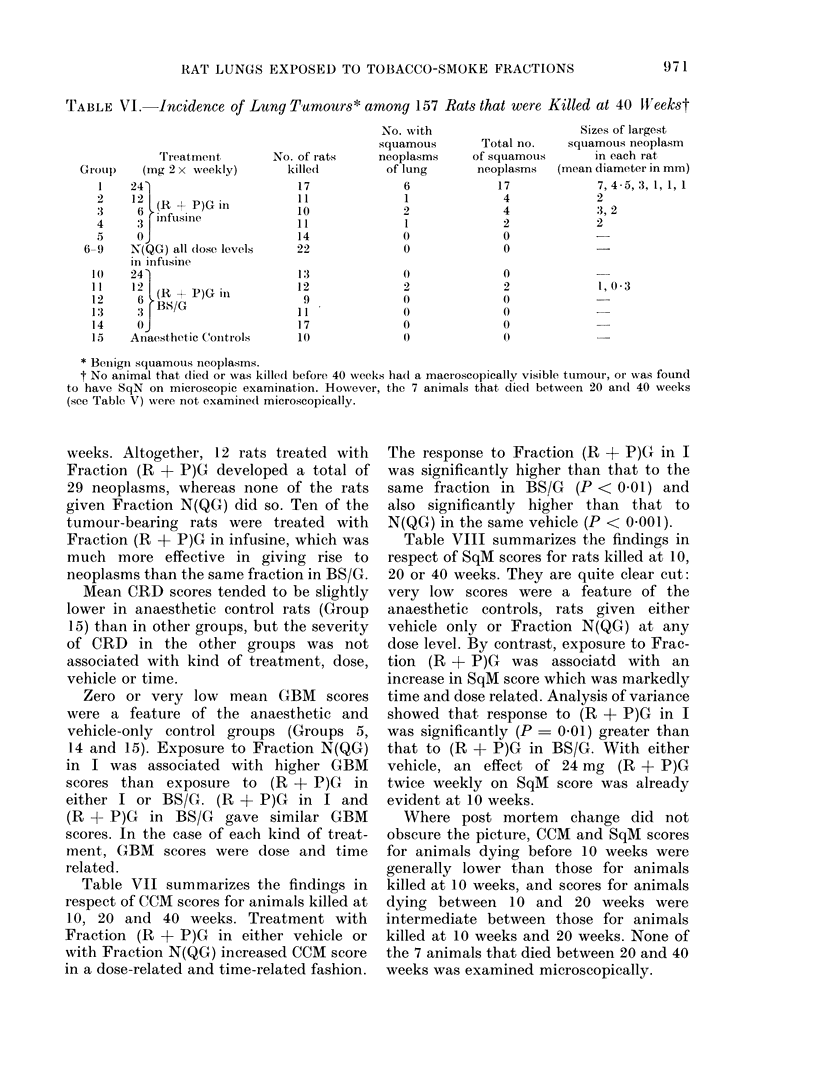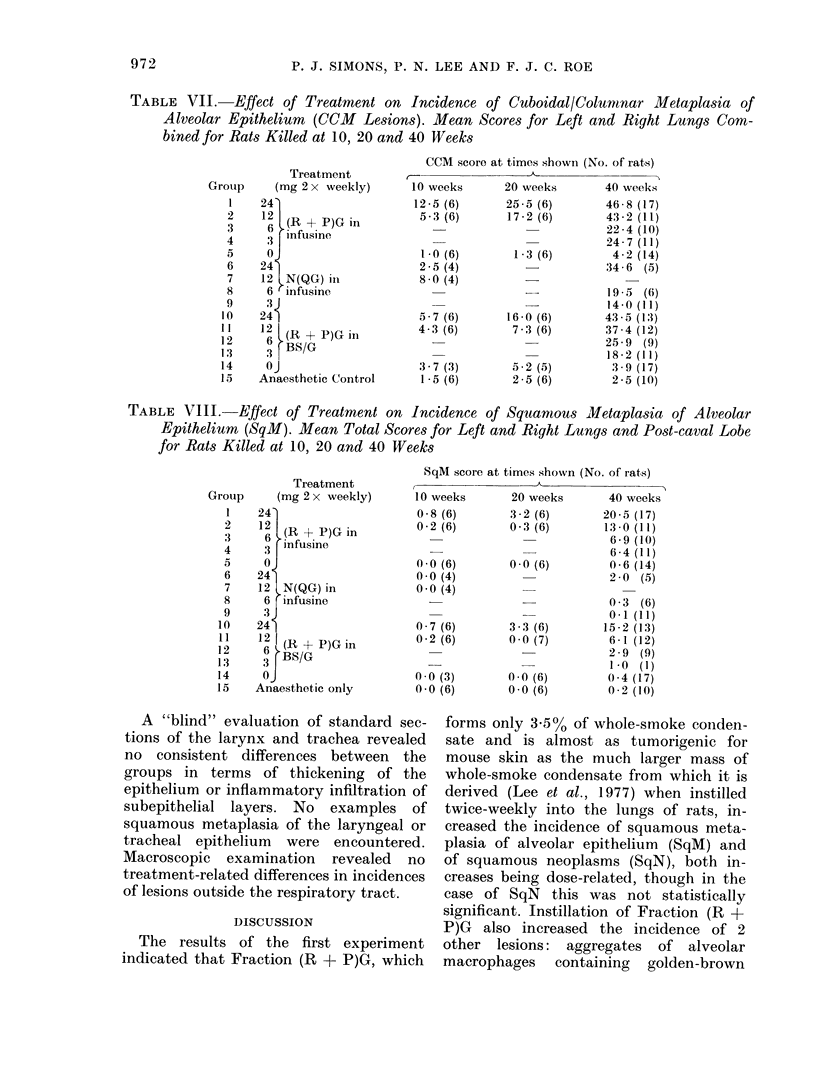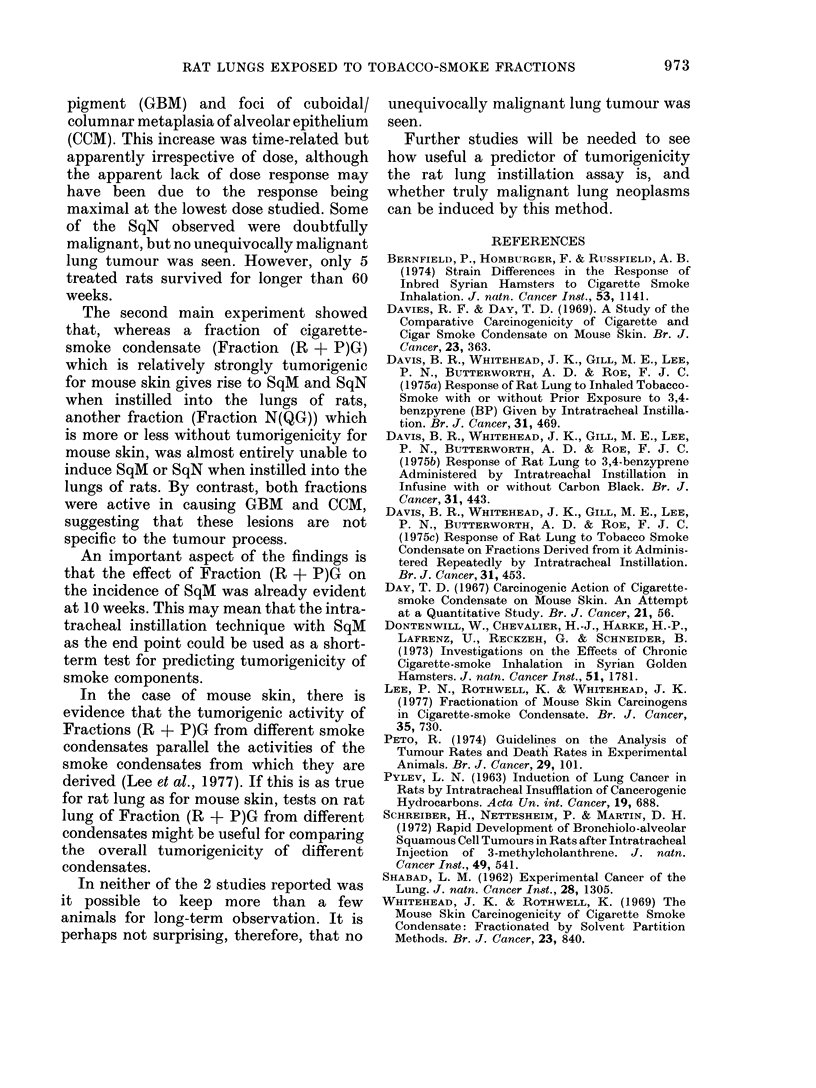# Squamous Lesions in the Lungs of Rats Exposed to Tobacco-smoke-condensate Fractions by Repeated Intratracheal Instillation

**DOI:** 10.1038/bjc.1978.141

**Published:** 1978-06

**Authors:** P. J. Simons, P. N. Lee, F. J. C. Roe

## Abstract

Twice-weekly intratracheal instillations in rats of up to 24 mg of Fraction (R + P)G suspended in either infusine (I) or buffered saline/gelatine (BS/G) gave rise to foci of squamous metaplasia of alveolar epithelium (SqM) and squamous neoplasms (SqN). Fraction (R + P)G, which is a fraction of cigarette-smoke condensate almost as tumorigenic for mouse skin as the nearly 30 × larger mass of condensate from which it is derived, could be given in this way for up to 40 weeks without excessive mortality or any marked effect on the rate of body-weight gain. By contrast, similar treatment with Fraction N(QG), a fraction having very low tumorigenic activity for mouse skin, induced no SqN and barely any excess of SqM over that induced by either vehicle alone.

The effects of Fraction (R + P)G on the incidence of SqM and SqN were both time and dose related, the effect on SqM incidence being already evident after 10 weeks of treatment. No SqN seen were unequivocally malignant, though, due to the design of the experiment, only 5 rats exposed to Fraction (R + P)G were observed more than 60 weeks after the start of the experiment.

Other changes in the lung, including aggregates of alveolar macrophages laden with golden-brown pigment (GBM) and foci of cuboidal/columnar metaplasia of alveolar epithelium (CCM), were frequently seen in response to both fractions. Fraction (R + P)G administered in I was more effective in causing SqM and SqN than the same fraction administered in BS/G. The implications of the findings are discussed, particularly the possibility that the intratracheal/instillation technique might be useful as a rapid bioassay for comparing the tumorigenicity of different cigarette-smoke condensates.


*Br. J. Cancer* (1978) **37**, 965

### SQUAMOUS LESIONS IN LUNGS OF RATS EXPOSED TO TOBACCO-SMOKE-CONDENSATE FRACTIONS BY REPEATED INTRATRACHEAL INSTILLATION

P. J. SIMONS*, P. N. LEE† AND F. J. C. ROE‡

*From the *Hazleton Laboratories Europe Ltd, Otley Road, Harrogate HG3 1PY, the †Tobacco Research Council, Glen House, Stag Place, London SW1E 5AG, and ‡4 Kings Road, Wimbledon, London SW19 8QN*

Received 3 February 1978 Accepted 29 March 1978

**Summary.—Twice-weekly intratracheal instillations in rats of up to 24 mg of Fraction (R + P)G suspended in either infusine (I) or buffered saline/gelatine (BS/G) gave rise to foci of squamous metaplasia of alveolar epithelium (SqM) and squamous neoplasms (SqN). Fraction (R + P)G, which is a fraction of cigarette-smoke condensate almost as tumorigenic for mouse skin as the nearly 30× larger mass of condensate from which it is derived, could be given in this way for up to 40 weeks without excessive mortality or any marked effect on the rate of body-weight gain. By contrast, similar treatment with Fraction N(QG), a fraction having very low tumorigenic activity for mouse skin, induced no SqN and barely any excess of SqM over that induced by either vehicle alone.**

**The effects of Fraction (R + P)G on the incidence of SqM and SqN were both time and dose related, the effect on SqM incidence being already evident after 10 weeks of treatment. No SqN seen were unequivocally malignant, though, due to the design of the experiment, only 5 rats exposed to Fraction (R + P)G were observed more than 60 weeks after the start of the experiment.**

**Other changes in the lung, including aggregates of alveolar macrophages laden with golden-brown pigment (GBM) and foci of cuboidal/columnar metaplasia of alveolar epithelium (CCM), were frequently seen in response to both fractions. Fraction (R + P)G administered in I was more effective in causing SqM and SqN than the same fraction administered in BS/G. The implications of the findings are discussed, particularly the possibility that the intratracheal/instillation technique might be useful as a rapid bioassay for comparing the tumorigenicity of different cigarette-smoke condensates.**

DESPITE several attempts, malignant tumours have not been produced in significant numbers in the lungs of experimental animals by exposing them to cigarette smoke (Dontenwill *et al.*, 1973; Bernfield *et al.*, 1974; Davis *et al.*, 1975*a*). Lung tumours have been readily induced by carcinogens administered by intratracheal instillation (Shabad, 1962; Pylev, 1963; Schreiber *et al.*, 1972; Davis *et al.*, 1975*b*). Davis *et al.* (1975*c*) also showed that the repeated fortnightly instillation of cigarette-smoke condensate (SWS) was associated with increased incidence of cuboidal columnar metaplasia (CCM) and squamous metaplasia (SqM), although such treatment failed to induce squamous neoplasms (SqN) in the lungs. Certain fractions of SWS known to be carcinogenic to mouse skin also produced CCM and SqM. In addition, Fraction P(SG), which contains most of the polycyclic aromatic hydrocarbons of cigarette smoke, produced a low incidence of benign or doubtfully malignant SqN, similar to those induced by the known carcinogen, benz(a)pyrene.

The object of the first experiment described in the present report was to determine whether rats will tolerate more frequent intratracheal instillations of SWS or smoke-condensate fractions and, if so, whether such treatment leads to the early development of SqN, or a high incidence of SqN, and/or to the development of SqN of undoubted malignancy.

The object of the second experiment was to compare the effects on rat lung of 2 fractions of tobacco-smoke condensate, one of which (Fraction (R + P)G) is far more tumorigenic for mouse skin than the other (Fraction N(QG)).

### MATERIALS AND METHODS

*Rats.*—Non-inbred Wistar specific-pathogen-free (SPF) rats obtained from Olac (Southern) Ltd, of Blackthorn, near Bicester, Oxon, were used for the 2 main experiments. Non-inbred Wistar SPF rats bred in our own laboratories (strain TRCL) were used for preliminary short-term toxicity trials.

In each experiment, female rats aged ~12 weeks were allocated by a non-selective process to the various treatment groups. Animals were housed in groups of 5 in solid-floor polypropylene cages with sawdust bedding, in natural daylight at 22 ± 3°C. They were fed Oxoid 41B laboratory animal diet and water *ad libitum*.

*Preparation of condensate and fractions.*—A single batch of plain cigarettes (TRC code T57) manufactured from a composite blend of flue-cured tobacco was used as the source for all condensate.

Smoke condensate was prepared by smoking cigarettes in the automatic smoking machine described by Day (1967). The standard smoking parameters used were: puff volume, 35 ml; puff duration, 2 s; puff frequency, 1 min; butt length, 20 mm. Smoke was collected in a glass trap cooled by immersion in acetone and crushed solid $CO_2$ (Davies and Day, 1969). It was stored at −29°C until used. Such a condensate was referred to as "stale whole-smoke condensate" (SWS).

The fractionation scheme has been described in detail (Whitehead and Rothwell, 1969; Lee *et al.*, 1977). The origins of the fractions used in these experiments were:

*Fractions G, (R + P)G and Q(G).*—Prepared from SWS by removal of the water-soluble materials and subsequent distribution of the water-insoluble residue (Fraction C) between 90% v/v aqueous methanol and cyclohexane (Fraction G). The caffeine-complexing material (Fraction (R + P)G) was removed by extracting fraction G dissolved in cyclohexane with a solution of caffeine in aqueous 90% formic acid to leave a residual fraction Q(G).

*Fractions K(QG) and L(QG).*—Prepared by distribution of Fraction Q(G) between cyclohexane and dimethyl sulphoxide (DMSO).

*Fraction HC(QG).*—Prepared by adsorption of Fraction Q(G) on an alumina column, followed by elution with petroleum ether and benzene.

*Fractions N(QG) and M(QG).*—Prepared by shaking a solution of Q(G) in benzene with de-activated silica gel. Fraction N(QG) was recovered from the benzene and Fraction M(QG) from the silica by elution with methanol.

*Chemicals.*—Dotriacontane was obtained from Koch-Light Labs. Ltd, and hexadecane from BHD Ltd. Both chemicals were recrystallized before use.

Infusine as described in Davis *et al.* (1975*a*) was used as the vehicle in most experiments. In the second main experiment phosphate-buffered saline (pH 7·4) containing 2% gelatine was used as one of the vehicles. This vehicle is referred to as "BS/G" in the Tables.

*Treatment and observation.*—The method for intratracheal instillation is described in Davis *et al.* (1975*a*). Animals were inspected daily for state of general health, clinically examined at the times of treatment, and weighed weekly. The techniques for post mortem examination and microscopic examination of tissues are described in Davis *et al.* (1975*a*).

*Histopathological evaluation.*—All rats that died or were killed during or at the end of the experiments were examined post mortem. Four standard 6 μm sections were prepared of the lungs of rats showing no localized lesions at necropsy. Additional sections were taken through macroscopically visible tumours, unless these were adequately represented in standard sections. Sections were stained with haematoxylin and eosin and scored in respect of 4 kinds of lesion: (i) aggregates of brown-pigment-laden alveolar macrophages (GBM); (ii) foci of cuboidal/columnar metaplasia of alveolar epithelium

(CCM); (iii) foci of squamous metaplasia of alveolar epithelium (SqM) and iv) squamous neoplasms (SqN). Slightly different scoring systems were used in the 2 experiments, and details are given separately in the text describing each experiment. Histopathological evaluations were "blind", in the sense that the pathologist (FJCR) was unaware of the treatment rats had received or of the week of the experiment in which they died or were killed.

*Statistical evaluation.*—Significance tests were carried out to assess between-group differences of 3 different parameters: (i) proportion of rats with a given lesion; (ii) mean number of lesions per rat and (iii) mean grade of lesion per rat. If the results being considered were based on rats dying in a single interval, chi-squared analysis was used for the first parameters and one-way analysis of variance for the second or third parameters. If a simultaneous assessment of results from rats dying in more than one interval was being made, Peto's method for incidental tumours (1974) was used for the first parameter, in order to correct for possible differences in survival between the groups. The other 2 parameters were analysed by unbalanced two-way analysis of variance, to give between-group differences "adjusted for time of death". All analyses of variance were carried out after a suitable variance-stabilizing transformation of the data. For the second parameters, which are counts, the square-root transformation $y = \sqrt{x}$ was used, while for the third parameters, which are scores out of a maximum possible ($M$) the transformation $y = \log_e[(x + 1)/(M + 1 - x)]$ was used.

Analysis of variance, this time of untransformed data, was also used to compare groups in terms of body weight and body-weight gain.

### EXPERIMENTAL DESIGN AND RESULTS

### *Experiment I*

Preliminary toxicity studies showed that rats would tolerate twice-weekly intratracheal instillations of 12 mg of Fraction (R + P)G in infusine, but not similar exposure to whole condensate (SWS) or Fraction G. The purpose of Exp. I was to see whether even higher doses of (R + P)G would be tolerated, and if so what effects such treatment would have.

Seventy rats were allocated non-selectively to 3 treatment groups, each of 18 animals, and 3 control groups, each of 4–6 animals. Rats in the first 3 groups received twice-weekly instillation of 12 mg, 24 mg or 48 mg of Fraction (R + P)G in infusine (total volume per instillation = 0·2 ml). One control group (6 rats) was similarly exposed to infusine only; another control group (6 rats) was anaesthetized twice-weekly but received no treatment; and the remaining control group (4 rats) received neither anaesthetic nor treatment. Treatment continued for 34 weeks, after which a proportion of the survivors were killed. The remaining rats were observed until the termination of the experiment at 84 weeks. The design of the study is

TABLE I.—*Survival of Rats Given up to 69 Twice-weekly Doses of Fraction (R + P)G Prepared from Cigarette TRC/57 by Intratracheal Instillation*

| Group | Treatment (2× weekly) | No. survivors at Week | | | | Killed at 34 weeks for histopathological examination | No. survivors at Week | | Killed at 84 weeks for histopathological examination |
|---|---|---|---|---|---|---|---|---|---|
| | | 0 | 10 | 20 | 34 | | 34+ | 60 | |
| 1 | 12 mg (R + P)G in infusine | 18 | 17 | 14 | 13 | 9 | 4 | 3 | 1 |
| 2 | 24 mg (R + P)G in infusine | 18 | 18 | 16 | 14 | 9 | 5 | 2 | 0 |
| 3 | 48 mg (R + P)G in infusine | 18 | 18 | 8 | 5 | 5 | 0 | 0 | 0 |
| 4 | Infusine only | 6 | 6 | 6 | 6 | 2 | 4 | 4 | 3 |
| 5 | Anaesthetic only | 6 | 5 | 5 | 4 | 2 | 2 | 1 | 0 |
| 6 | None | 4 | 4 | 4 | 4 | 0 | 4 | 4 | 4 |
| | | 70 | 68 | 53 | 46 | 27 | 19 | 14 | 8 |

TABLE II.—*Mean Initial Body Weight and Body-weight Gain*

| Group | Treatment (2× weekly) | | Initial body weight | Weight gain by week 4 | 8 | 16 | 32 |
|---|---|---|---|---|---|---|---|
| 1 | 12 mg (R + P)G | Mean | 229·9 | 17·2 | 24·5 | 43·9 | 55·4 |
| | in infusine | s.e. | 3·6 | 1·6 | 2·0 | 2·8 | 4·3 |
| 2 | 24 mg (R + P)G | Mean | 228·1 | 6·0 | 24·2 | 41·4 | 57·5 |
| | in infusine | s.e. | 2·8 | 2·0 | 2·4 | 3·7 | 4·7 |
| 3 | 48 mg (R + P)G | Mean | 220·1 | 4·2 | 19·8 | 40·4 | 44·4 |
| | in infusine | s.e. | 7·4 | 1·6 | 2·7 | 2·0 | 3·9 |
| 4 | Infusine only | Mean | 239·5 | 11·0 | 28·2 | 51·8 | 65·5 |
| | | s.e. | 6·4 | 2·9 | 6·1 | 5·4 | 3·8 |
| 5 | Anaesthetic only | Mean | 242·3 | 10·0 | 35·0 | 51·0 | 73·5 |
| | | s.e. | 4·3 | 2·4 | 5·4 | 5·2 | 8·3 |
| 6 | None | Mean | 228·2 | 4·6 | 28·2 | 36·0 | 61·6 |
| | | s.e. | 8·8 | 2·7 | 3·5 | 7·1 | 7·6 |

TABLE III.—*Macroscopically Visible Lung Tumours (T) and Microscopically Observed Squamous Neoplasms (SqN) in Rats Examined at Post Mortem*†

| Group | Treatment (2× weekly) | Examined at post mortem‡ | No. rats with T O* | T E | SqN O | SqN E | Mean no. SqN/rat |
|---|---|---|---|---|---|---|---|
| 1 | 12 mg (R + P)G in infusine | 17 | 3 | 4·53 | 7 | 9·62 | 0·47 |
| 2 | 24 mg (R + P)G in infusine | 15 | 5 | 4·45 | 11 | 9·59 | 1·80 |
| 3 | 48 mg (R + P)G in infusine | 15 | 4 | 3·02 | 7 | 5·80 | 2·07 |
| 4, 5, & 6 | Combined control groups | 6 | 0 | — | 0 | — | 0 |

* O = Number observed; E = Number expected if the effects of the 3 dose levels had been the same, after allowing for survival differences (see text).

† All macroscopically observed tumours were found on microscopic examination to be squamous neoplasms. However several small squamous neoplasms not observed at necropsy were discovered on microscopic examination of the lungs.

‡ 7 treated rats in groups 1–3 were too autolysed for assessment and only 6 control rats (groups 4–6) were assessed. Otherwise the Table refers to all rats in the experiment, irrespective of when they died or were killed.

evident from Table I, which also gives survival data.

Table I shows that twice-weekly intratracheal instillations of 12 mg or 24 mg, but not 48 mg, (R + P)G were well tolerated in terms of survival. As shown in Table II, the mean body weight of rats in all the groups increased during the study. Analysis of variance showed that, though there were marked differences between the 6 groups in weight gain in the first 4 weeks ($P < 0{\cdot}001$) due mainly to a marked dose-response trend, these were not subsequently statistically significant. However the (R + P)G-treated groups had consistently lower weight gains than the infusine-only or anaesthetic-only groups. The behaviour of the untreated group was surprisingly erratic.

Tables III and IV summarize the findings in terms of tumours observed macroscopically at necropsy and GBM, SqM and SqN scores derived from a "blind" histopathological evaluation of sections from 53 of the 70 rats. The scoring system used in this study was as follows. The available sections of the lungs of each rat were given an arbitrary GBM score of 0–4 according to an overall assessment of the frequency of aggregates of pigment-laden macrophages. Arbitrary CCM scores on a 0–4 scale and SqM on a 0–3 scale were arrived at in a similar way. The squamous neoplasms encountered consisted of large masses of keratin surrounded by a narrow zone of squamous epithelium. Apart from their large size and excessive keratin component, the lesions were difficult to distinguish from squamous metaplasia. Undoubtedly benign lesions were graded "4" and lesions which showed possible evidence of extension into surrounding

TABLE IV.—*Microscopic Findings in Rats Killed at* 34 *Weeks*

| Group | Treatment (2× weekly) | No. rats | Mean grade of GBM | CCM | SqM |
|---|---|---|---|---|---|
| 1 | 12 mg (R + P)G in infusine | 9 | 3·08 | 3·03 | 1·58 |
| 2 | 24 mg (R + P)G in infusine | 9 | 3·36 | 3·53 | 2·26 |
| 3 | 48 mg (R + P)G in infusine | 5 | 2·70 | 3·27 | 2·49 |
| 4, 5 & 6 | Combined control groups | 4 | 0 | 0·50 | 0 |

lung tissue were graded "5". These grades were a direct extension of the grades 0–3 used for SqM lesions.

The results in Table III show clearly that tumours and SqN were related to the (R + P)G treatment, none being seen in any of the 6 control rats examined. After taking between-group survival differences into account, no statistically significant side response was found in the numbers with either tumour or SqN, though there was some indication of a positive trend, as indicated by the excess in the highest dose levels in the observed numbers (O) over those expected (E) under the assumption that dose does not influence incidence. There was, however, clear evidence of a significant dose-related effect on the mean number of SqN per rat, the mean number in Group 1 being very significantly ($P <$ 0·001) less than that in the other 2 groups.

As all 3 lesions studied in Table IV are strongly time-related, the Table simplifies the comparison of the groups by considering only those rats killed at Week 34. Though (R + P)G itself had a marked effect on all 3 types of lesion, there was no clear evidence of a dose relationship in the effects of treatment on mean GBM and CCM scores. There was, however, a very highly significant dose-related effect on mean SqM score ($P <$ 0·01).

Full examination of other organs at necropsy revealed no evidence of metastasis from any of the lung tumours.

*Experiment II*

The purpose of this experiment was to compare the response of the rat lung to a fraction of smoke condensate known to be tumorigenic for mouse-skin ((R + P)G) with that to one known to have little or no tumorigenic effect. At the same time it was proposed to compare the influence on response of 2 different vehicles, infusine (I) and buffered saline/gelatine (BS/G).

Preliminary toxicity studies were undertaken to identify a suitable fraction of smoke condensate which was not tumorigenic for mouse-skin and which would be tolerated by rats exposed to it by intratracheal instillation. Short-term studies indicated that Fractions K(QG), L(QG), M(QG) and HC(QG), and also dotriacontane, hexadecane and liquid paraffin (BPC), were too toxic, but that Fraction N(QG) might be sufficiently well-tolerated for the purposes of the proposed study.

A preliminary short-term study was also carried out to see whether the frequency of dosing could be increased from twice to 3× weekly without an adverse effect on survival of rats given 12 or 24 mg of (R + P)G. The results showed that mortality with 3× weekly treatment was excessive and it was, therefore, decided to continue with 2× weekly application in Experiment II.

The design of Experiment II, involving a comparison of the effects of Fractions (R + P)G and N(QG) with I or BS/G as vehicle is evident from Table V. The Table shows that 309 rats were allocated non-selectively to 15 groups, of which 46 died before Week 10, 47 from 9 different groups were killed at 10 weeks, 10 died between 10 and 20 weeks, 42 from 7 different groups were killed at 20 weeks, 7 died between 20 and 40 weeks, and the remaining 157 were killed at 40 weeks. From these figures it can be seen that Fraction N(QG) at the dose levels 24 mg or 12 mg twice weekly in I caused more premature deaths than any other treatment, and that Fraction (R + P)G at the dose level of 24 mg twice weekly in BS/G

TABLE V.—*Experiment II: Design and Survival*

| Group | Treatment (mg 2× weekly) for up to 40 weeks | No. of rats | Died before Week 10 | No. killed at 10 weeks | Died between 10 and 20 weeks | No. killed at 20 weeks | Died between 20 and 40 weeks | No. killed at 40 weeks |
|---|---|---|---|---|---|---|---|---|
| 1 | 24 (R + P)G in infusine | 34 | 4 | 6 | 1 | 6 | 0 | 17 |
| 2 | 12 | 24 | 1 | 6 | 0 | 6 | 0 | 11 |
| 3 | 6 | 12 | 2 | 0 | 0 | 0 | 0 | 10 |
| 4 | 3 | 12 | 1 | 0 | 0 | 0 | 0 | 11 |
| 5 | 0 | 29 | 2 | 6 | 0 | 6 | 1 | 14 |
| 6 | 24 N(QG) in infusine | 23 | 11 | 4 | 3 | 0 | 0 | 5 |
| 7 | 12 | 20 | 11 | 4 | 3 | 0 | 2 | 0 |
| 8 | 6 | 10 | 1 | 0 | 2 | 0 | 1 | 6 |
| 9 | 3 | 12 | 1 | 0 | 0 | 0 | 0 | 11 |
| 10 | 24 (R + P)G in BS/G | 35 | 7 | 6 | 1 | 6 | 2 | 13 |
| 11 | 12 | 24 | 0 | 6 | 0 | 6 | 0 | 12 |
| 12 | 6 | 10 | 1 | 0 | 0 | 0 | 0 | 9 |
| 13 | 3 | 12 | 1 | 0 | 0 | 0 | 0 | 11 |
| 14 | 0 | 30 | 3 | 3 | 0 | 6 | 1 | 17 |
| 15 | Anaesthetic Control | 22 | 0 | 6 | 0 | 6 | 0 | 10 |
| | | 309 | 46 | 47 | 10 | 42 | 7 | 157 |

caused more deaths than when I was the vehicle (10 *vs* 5).

There were no striking differences between groups in rate of body-weight gain during the experiment, except that the anaesthetic control group (Group 15) put on more weight (60 g) between the 4th and 40th weeks of the experiment than any other group (range 32–50 g).

Groups were compared in respect of (i) incidence of macroscopically visible lung tumours, (ii) severity of chronic respiratory diseases (CRD), (iii) incidence of aggregates of GBM, (iv) incidence of CCM lesions, (v) incidence of SqM lesions and (vi) microscopically observed SqN. Standard sections of the left and right lungs and post-caval lobe (*i.e.* 3 sections in all) were evaluated "blind" in respect of parameters (ii)–(vi). CRD was assessed on the scale 0–4 (0 = none, 1 = minimal, 2 = slight, 3 = of moderate severity, 4 = severe.) Various kinds of spontaneous lung disease were taken into account in arriving at a score for each animal; *e.g.* aggregates of lymphocytes and plasma cells around main airways and blood vessels, interstitial pneumonitis, focal granulomatous lesions, focal consolidation and bronchopneumonia. The score reflected both the severity and extent of disease. Scores for aggregates of GBM were allocated as follows: 0 = none, 1 = occasional clusters, 2 = moderately frequent clusters, 3 = frequent clusters, 4 = very numerous clusters, 5 = extensive masses of pigment-laden macrophages. Scores for CCM were based on the total numbers of lesions observed in complete scan of the left and right lung section up to a maximum of 25 lesions per lung. Large foci of CCM were counted 2×, 3×, 4× *etc.*, according to the number of low-power fields upon which they encroached. Scores for SqM were arrived at in a directly comparable way to that used for CCM. All the squamous neoplasms (SqN) seen were undoubtedly benign and were graded "4". The mean diameters of SqN were recorded.

Only 4 rats had lung tumours that were visible at necropsy. All were among groups treated with (R + P)G in infusine that survived for the full 40 weeks of the experiment. Further lung tumours lying deep in the lung tissue were seen when the lungs were trimmed after fixation. In addition, several small SqN were discovered for the first time during the microscopic examination of the lungs. No animal that died or was killed before the 40th week of the experiment was found to have an SqN, though the 7 dying between 20 and 40 weeks were not examined microscopically. Table VI shows the incidence of SqN among the 157 rats that were killed at 40

TABLE VI.—*Incidence of Lung Tumours* among* 157 *Rats that were Killed at* 40 *Weeks*†

| Group | Treatment (mg 2× weekly) | No. of rats killed | No. with squamous neoplasms of lung | Total no. of squamous neoplasms | Sizes of largest squamous neoplasm in each rat (mean diameter in mm) |
|---|---|---|---|---|---|
| 1 | 24 (R + P)G in infusine | 17 | 6 | 17 | 7, 4·5, 3, 1, 1, 1 |
| 2 | 12 (R + P)G in infusine | 11 | 1 | 4 | 2 |
| 3 | 6 (R + P)G in infusine | 10 | 2 | 4 | 3, 2 |
| 4 | 3 (R + P)G in infusine | 11 | 1 | 2 | 2 |
| 5 | 0 (R + P)G in infusine | 14 | 0 | 0 | — |
| 6–9 | N(QG) all dose levels in infusine | 22 | 0 | 0 | — |
| 10 | 24 (R + P)G in BS/G | 13 | 0 | 0 | — |
| 11 | 12 (R + P)G in BS/G | 12 | 2 | 2 | 1, 0·3 |
| 12 | 6 (R + P)G in BS/G | 9 | 0 | 0 | — |
| 13 | 3 (R + P)G in BS/G | 11 | 0 | 0 | — |
| 14 | 0 (R + P)G in BS/G | 17 | 0 | 0 | — |
| 15 | Anaesthetic Controls | 10 | 0 | 0 | — |

* Benign squamous neoplasms.
† No animal that died or was killed before 40 weeks had a macroscopically visible tumour, or was found to have SqN on microscopic examination. However, the 7 animals that died between 20 and 40 weeks (see Table V) were not examined microscopically.

weeks. Altogether, 12 rats treated with Fraction (R + P)G developed a total of 29 neoplasms, whereas none of the rats given Fraction N(QG) did so. Ten of the tumour-bearing rats were treated with Fraction (R + P)G in infusine, which was much more effective in giving rise to neoplasms than the same fraction in BS/G.

Mean CRD scores tended to be slightly lower in anaesthetic control rats (Group 15) than in other groups, but the severity of CRD in the other groups was not associated with kind of treatment, dose, vehicle or time.

Zero or very low mean GBM scores were a feature of the anaesthetic and vehicle-only control groups (Groups 5, 14 and 15). Exposure to Fraction N(QG) in I was associated with higher GBM scores than exposure to (R + P)G in either I or BS/G. (R + P)G in I and (R + P)G in BS/G gave similar GBM scores. In the case of each kind of treatment, GBM scores were dose and time related.

Table VII summarizes the findings in respect of CCM scores for animals killed at 10, 20 and 40 weeks. Treatment with Fraction (R + P)G in either vehicle or with Fraction N(QG) increased CCM score in a dose-related and time-related fashion. The response to Fraction (R + P)G in I was significantly higher than that to the same fraction in BS/G ($P < 0{\cdot}01$) and also significantly higher than that to N(QG) in the same vehicle ($P < 0{\cdot}001$).

Table VIII summarizes the findings in respect of SqM scores for rats killed at 10, 20 or 40 weeks. They are quite clear cut: very low scores were a feature of the anaesthetic controls, rats given either vehicle only or Fraction N(QG) at any dose level. By contrast, exposure to Fraction (R + P)G was associatd with an increase in SqM score which was markedly time and dose related. Analysis of variance showed that response to (R + P)G in I was significantly ($P = 0{\cdot}01$) greater than that to (R + P)G in BS/G. With either vehicle, an effect of 24 mg (R + P)G twice weekly on SqM score was already evident at 10 weeks.

Where post mortem change did not obscure the picture, CCM and SqM scores for animals dying before 10 weeks were generally lower than those for animals killed at 10 weeks, and scores for animals dying between 10 and 20 weeks were intermediate between those for animals killed at 10 weeks and 20 weeks. None of the 7 animals that died between 20 and 40 weeks was examined microscopically.

TABLE VII.—*Effect of Treatment on Incidence of Cuboidal/Columnar Metaplasia of Alveolar Epithelium (CCM Lesions). Mean Scores for Left and Right Lungs Combined for Rats Killed at* 10, 20 *and* 40 *Weeks*

| Group | Treatment (mg 2× weekly) | | CCM score at times shown (No. of rats) | | |
|---|---|---|---|---|---|
| | | | 10 weeks | 20 weeks | 40 weeks |
| 1 | 24 | (R + P)G in infusine | 12·5 (6) | 25·5 (6) | 46·8 (17) |
| 2 | 12 | | 5·3 (6) | 17·2 (6) | 43·2 (11) |
| 3 | 6 | | — | — | 22·4 (10) |
| 4 | 3 | | — | — | 24·7 (11) |
| 5 | 0 | | 1·0 (6) | 1·3 (6) | 4·2 (14) |
| 6 | 24 | N(QG) in infusine | 2·5 (4) | — | 34·6 (5) |
| 7 | 12 | | 8·0 (4) | — | — |
| 8 | 6 | | — | — | 19·5 (6) |
| 9 | 3 | | — | — | 14·0 (11) |
| 10 | 24 | (R + P)G in BS/G | 5·7 (6) | 16·0 (6) | 43·5 (13) |
| 11 | 12 | | 4·3 (6) | 7·3 (6) | 37·4 (12) |
| 12 | 6 | | — | — | 25·9 (9) |
| 13 | 3 | | — | — | 18·2 (11) |
| 14 | 0 | | 3·7 (3) | 5·2 (5) | 3·9 (17) |
| 15 | Anaesthetic Control | | 1·5 (6) | 2·5 (6) | 2·5 (10) |

TABLE VIII.—*Effect of Treatment on Incidence of Squamous Metaplasia of Alveolar Epithelium (SqM). Mean Total Scores for Left and Right Lungs and Post-caval Lobe for Rats Killed at* 10, 20 *and* 40 *Weeks*

| Group | Treatment (mg 2× weekly) | | SqM score at times shown (No. of rats) | | |
|---|---|---|---|---|---|
| | | | 10 weeks | 20 weeks | 40 weeks |
| 1 | 24 | (R + P)G in infusine | 0·8 (6) | 3·2 (6) | 20·5 (17) |
| 2 | 12 | | 0·2 (6) | 0·3 (6) | 13·0 (11) |
| 3 | 6 | | — | — | 6·9 (10) |
| 4 | 3 | | — | — | 6·4 (11) |
| 5 | 0 | | 0·0 (6) | 0·0 (6) | 0·6 (14) |
| 6 | 24 | N(QG) in infusine | 0·0 (4) | — | 2·0 (5) |
| 7 | 12 | | 0·0 (4) | — | — |
| 8 | 6 | | — | — | 0·3 (6) |
| 9 | 3 | | — | — | 0·1 (11) |
| 10 | 24 | (R + P)G in BS/G | 0·7 (6) | 3·3 (6) | 15·2 (13) |
| 11 | 12 | | 0·2 (6) | 0·0 (7) | 6·1 (12) |
| 12 | 6 | | — | — | 2·9 (9) |
| 13 | 3 | | — | — | 1·0 (1) |
| 14 | 0 | | 0·0 (3) | 0·0 (6) | 0·4 (17) |
| 15 | Anaesthetic only | | 0·0 (6) | 0·0 (6) | 0·2 (10) |

A "blind" evaluation of standard sections of the larynx and trachea revealed no consistent differences between the groups in terms of thickening of the epithelium or inflammatory infiltration of subepithelial layers. No examples of squamous metaplasia of the laryngeal or tracheal epithelium were encountered. Macroscopic examination revealed no treatment-related differences in incidences of lesions outside the respiratory tract.

### DISCUSSION

The results of the first experiment indicated that Fraction (R + P)G, which forms only 3·5% of whole-smoke condensate and is almost as tumorigenic for mouse skin as the much larger mass of whole-smoke condensate from which it is derived (Lee *et al.*, 1977) when instilled twice-weekly into the lungs of rats, increased the incidence of squamous metaplasia of alveolar epithelium (SqM) and of squamous neoplasms (SqN), both increases being dose-related, though in the case of SqN this was not statistically significant. Instillation of Fraction (R + P)G also increased the incidence of 2 other lesions: aggregates of alveolar macrophages containing golden-brown pigment (GBM) and foci of cuboidal/columnar metaplasia of alveolar epithelium (CCM). This increase was time-related but apparently irrespective of dose, although the apparent lack of dose response may have been due to the response being maximal at the lowest dose studied. Some of the SqN observed were doubtfully malignant, but no unequivocally malignant lung tumour was seen. However, only 5 treated rats survived for longer than 60 weeks.

The second main experiment showed that, whereas a fraction of cigarette-smoke condensate (Fraction (R + P)G) which is relatively strongly tumorigenic for mouse skin gives rise to SqM and SqN when instilled into the lungs of rats, another fraction (Fraction N(QG)) which is more or less without tumorigenicity for mouse skin, was almost entirely unable to induce SqM or SqN when instilled into the lungs of rats. By contrast, both fractions were active in causing GBM and CCM, suggesting that these lesions are not specific to the tumour process.

An important aspect of the findings is that the effect of Fraction (R + P)G on the incidence of SqM was already evident at 10 weeks. This may mean that the intratracheal instillation technique with SqM as the end point could be used as a short-term test for predicting tumorigenicity of smoke components.

In the case of mouse skin, there is evidence that the tumorigenic activity of Fractions (R + P)G from different smoke condensates parallel the activities of the smoke condensates from which they are derived (Lee *et al.*, 1977). If this is as true for rat lung as for mouse skin, tests on rat lung of Fraction (R + P)G from different condensates might be useful for comparing the overall tumorigenicity of different condensates.

In neither of the 2 studies reported was it possible to keep more than a few animals for long-term observation. It is perhaps not surprising, therefore, that no unequivocally malignant lung tumour was seen.

Further studies will be needed to see how useful a predictor of tumorigenicity the rat lung instillation assay is, and whether truly malignant lung neoplasms can be induced by this method.

### REFERENCES

BERNFIELD, P., HOMBURGER, F. & RUSSFIELD, A. B. (1974) Strain Differences in the Response of Inbred Syrian Hamsters to Cigarette Smoke Inhalation. *J. natn. Cancer Inst.*, **53**, 1141.

DAVIES, R. F. & DAY, T. D. (1969). A Study of the Comparative Carcinogenicity of Cigarette and Cigar Smoke Condensate on Mouse Skin. *Br. J. Cancer*, **23**, 363.

DAVIS, B. R., WHITEHEAD, J. K., GILL, M. E., LEE, P. N., BUTTERWORTH, A. D. & ROE, F. J. C. (1975*a*) Response of Rat Lung to Inhaled Tobacco-Smoke with or without Prior Exposure to 3,4-benzpyrene (BP) Given by Intratracheal Instillation. *Br. J. Cancer*, **31**, 469.

DAVIS, B. R., WHITEHEAD, J. K., GILL, M. E., LEE, P. N., BUTTERWORTH, A. D. & ROE, F. J. C. (1975*b*) Response of Rat Lung to 3,4-benzyprene Administered by Intratreachal Instillation in Infusine with or without Carbon Black. *Br. J. Cancer*, **31**, 443.

DAVIS, B. R., WHITEHEAD, J. K., GILL, M. E., LEE, P. N., BUTTERWORTH, A. D. & ROE, F. J. C. (1975*c*) Response of Rat Lung to Tobacco Smoke Condensate on Fractions Derived from it Administered Repeatedly by Intratracheal Instillation. *Br. J. Cancer*, **31**, 453.

DAY, T. D. (1967) Carcinogenic Action of Cigarette-smoke Condensate on Mouse Skin. An Attempt at a Quantitative Study. *Br. J. Cancer*, **21**, 56.

DONTENWILL, W., CHEVALIER, H.-J., HARKE, H.-P., LAFRENZ, U., RECKZEH, G. & SCHNEIDER, B. (1973) Investigations on the Effects of Chronic Cigarette-smoke Inhalation in Syrian Golden Hamsters. *J. natn. Cancer Inst.*, **51**, 1781.

LEE, P. N., ROTHWELL, K. & WHITEHEAD, J. K. (1977) Fractionation of Mouse Skin Carcinogens in Cigarette-smoke Condensate. *Br. J. Cancer*, **35**, 730.

PETO, R. (1974) Guidelines on the Analysis of Tumour Rates and Death Rates in Experimental Animals. *Br. J. Cancer*, **29**, 101.

PYLEV, L. N. (1963) Induction of Lung Cancer in Rats by Intratracheal Insufflation of Cancerogenic Hydrocarbons. *Acta Un. int. Cancer*, **19**, 688.

SCHREIBER, H., NETTESHEIM, P. & MARTIN, D. H. (1972) Rapid Development of Bronchiolo-alveolar Squamous Cell Tumours in Rats after Intratracheal Injection of 3-methylcholanthrene. *J. natn. Cancer Inst.*, **49**, 541.

SHABAD, L. M. (1962) Experimental Cancer of the Lung. *J. natn. Cancer Inst.*, **28**, 1305.

WHITEHEAD, J. K. & ROTHWELL, K. (1969) The Mouse Skin Carcinogenicity of Cigarette Smoke Condensate: Fractionated by Solvent Partition Methods. *Br. J. Cancer*, **23**, 840.